# Aplysinopsins - Marine Indole Alkaloids: Chemistry, Bioactivity and Ecological Significance

**DOI:** 10.3390/md7020166

**Published:** 2009-05-19

**Authors:** Dobroslawa Bialonska, Jordan K. Zjawiony

**Affiliations:** 1 Department of Pharmacognosy, School of Pharmacy, University of Mississippi, University, MS 38677-1848, USA; E-Mail: dbialons@olemiss.edu; 2 Research Institute of Pharmaceutical Sciences, University of Mississippi, University, MS 38677-1848, USA; 3 Institute of Environmental Sciences, Jagiellonian University, Gronostajowa 7, 30–387 Krakow, Poland

**Keywords:** aplysinopsins, natural source, stereochemistry, bioactivity, ecological functions

## Abstract

Aplysinopsins are tryptophan-derived marine natural products isolated from numerous genera of sponges and scleractinian corals, as well as from one sea anemone and one nudibranch. Aplysinopsins are widely distributed in the Pacific, Indonesia, Caribbean, and Mediterranean regions. Up to date, around 30 analogues occurring in Nature have been reported. Natural aplysinopsins differ in the bromination pattern of the indole ring, variation in the structure of the C ring, including the number and position of *N*-methylation, the presence and configuration of the C-8-C-1′ double bond, and the oxidation state of the 2-aminoimidazoline fragment. Aplysinopsins can also occur in the form of dimers. This review summarizes 30 years’ research on aplysinopsins. The origin, isolation sources, chemistry, bioactivity, and ecological functions of aplysinopsins are comprehensively reviewed.

## 1. Introduction

Aplysinopsin (**1**) was isolated for the first time by Kazlauskas *et al.* [1] as the major metabolite of eight Indo-Pacific sponge species, representatives of the genera *Thorecta* (later assigned as the separate *Aplysinopsis* genera). Since that time, aplysinopsin and its derivatives have been reported in many other marine organisms from various geographic locations (Table 1). Aplysinopsin-type compounds have been found in sponges of the Caribbean: *Verongia spengelli* [2], *Dercitus* sp. [3], *Smenospongia aurea* [4–6], *Verongula rigida* [7]; the Mediterranean Sea: *Dictyoceratida* sp. [8]; as well as in the Indo-Pacific region: *Aplysinopsis reticulata* [1,9], *Aplysina* sp. [10], *Hyrtios erecta* [11], *Smenospongia* sp. [12], and *Thorectandra* sp. [12].

Aplysinopsins, initially believed to be produced exclusively by Porifera, have been also detected in many anthozoan scleractinian corals. First reported from *Astroides calycularis* collected in the Mediterranean Sea [13], later aplysinopsins were described also from *Leptopsammia pruvoti* in the same geographic location [14]. In addition, aplysinopsins were isolated from corals of Indo-Pacific reefs: *Tubastraea coccinea* [15], *Tubastraea aurea* [16], *Dendrophyllia* sp. [17], *Tubastraea faulkneri* [18], and *Tubastraea* sp. [16,19].

Interestingly, aplysinopsin-type compounds have also been described for the mollusc *Phestilla melanobrachia,* feeding exclusively on the coral *Tubastrea coccinea* [15], as well as for the sea anemone *Radianthus kuekenthali.* In the last case aplysinopsins play a signaling part in anemone-anemone fish symbiosis [20].

**Figure 1:**
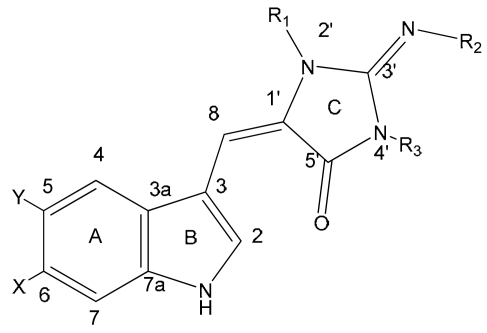
Structures of aplysinopsins **1–11**. General formula of aplysinopsin derivatives shown as *E*-isomer dominant in nature (imino-tautomer of ring C is shown). **Table:** 

**Figure 2:**
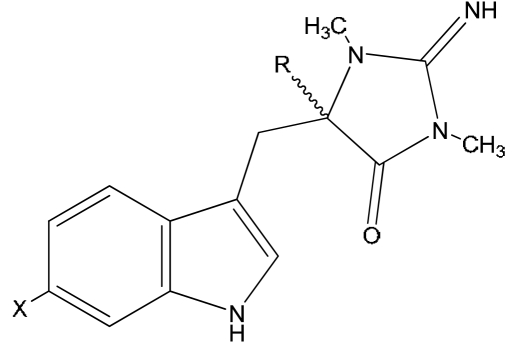
Structures of aplysinopsins **12–16**. **Table:** 

**Figure 3:**
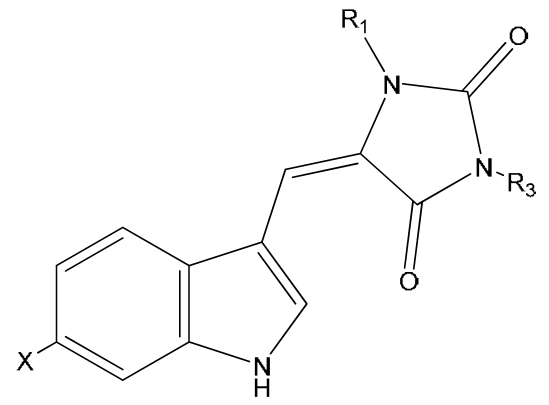
Structures of aplysinopins **17–20**. **Table:** 

**Figure 4:**
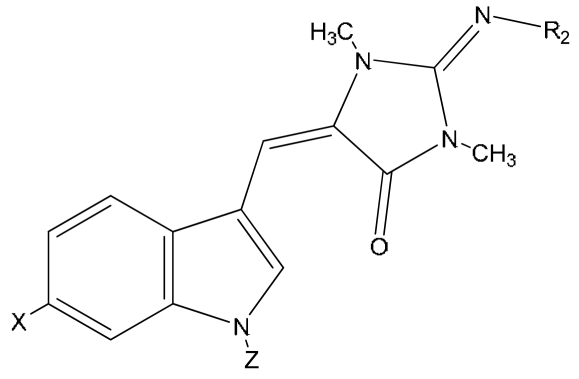
Structures of aplysinopsins **21–23**. **Table:** 

Natural derivatives of aplysinopsin differ in the bromination pattern of the indole moiety (Figure 1), variation in the structure of the C ring, including its oxidation state (Figure 3) and number and position of *N*-methylation (Figure 1), the presence and absence of the C-8-C-1′ double bond (Figure 2), and the stereochemistry. Finally, aplysinopsins in the form of dimers have also been reported (Figure 4).

Most of the 1,600 known naturally occurring organobromine compounds are found in marine organisms [21]. The presence of an unidentified monobromo analogue of aplysinopsin had been reported by Kazlauskas *et al.* in the first report on aplysinopsin [1], but owing to the insufficient amount available the authors were not able to isolate that compound or elucidate its structure. Later, brominated aplysinopsins have been described for sponges, corals, anemone and a mollusk. In fact, around a half of all isolated aplysinopsin-like compounds are halogenated with bromine. Interestingly, in almost all brominated aplysinopsins isolated from Nature so far, halogenation occurred at the 6 position of the indole moiety, with the exception of only one compound, 5,6-dibromo-2′-demethylaplysinopsin (**10**) brominated at both positions 5 and 6 (Table 1).

The structural diversity of natural aplysinopsins includes also variations in number and positions of methyl groups in ring C, including compounds with one (**3**,**4**,**6**,**10**), two (**1**,**2**,**5**,**8**,**9**) and three methyl groups (**7**). An aplysinopsin analog having higher alkyl groups in ring C (**11**) is also known (Figure 1). Another group of analogs (Figure 2) consists of compounds with a single C-8 - C-1′ bond, e.g. 1′,8-dihydroaplysinopsin (**12**) and its 6-bromo derivative (**13**). In addition, derivatives of **13** hydroxylated, methoxylated, and ethoxylated at C-1′were isolated by Segraves and Crews [12] from Indo-Pacific sponges.

Examples of derivatives with different levels of oxidation of the 2-aminoimidazoline moiety include 3′-deimino-3′-oxoaplysinopsin (**17**) and 3′-deimino-2′,4′-bis(demethyl)-3′-oxoaplysinopsin (**19**), as well as their brominated analogues (**18** and **20**) (Figure 3).

Aplysinopsins substituted at the nitrogen atom of the indole ring were also reported, e.g. compound **23** isolated from Japanese marine sponge [10], and compounds **21**, **22** from the Mediterranean anthozoan *Astroides calycularis* (Figure 4) [13].

**Figure 5:**
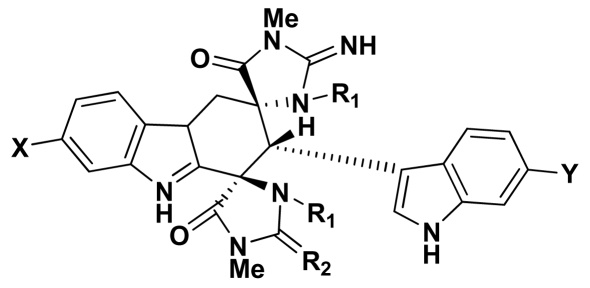
Dimers of aplysinopsin-type compounds. Compound **24:** X = Br, Y = Br, R_1_ = H, R_2_ = NH Compound **25:** X = H, Y = Br, R_1_ = CH_3_, R_2_ = NH Compound **26:** X = H, Y = H, R_1_ = CH_3_, R_2_ = NH Compound **27:** X = H, Y = H, R_1_ = CH_3_, R_2_ = O

Finally, aplysinopsin dimers were detected in *Tubastraea* sp. a stony coral from the Indo-Pacific area. This includes a dimer of 6-bromo-2′-de-*N*-methylaplysinopsin (**24**) [18], and three other analogues (**25**–**27**) isolated by Iwagawa et al (Figure 5) [19].

After the isolation of aplysinopsin from a marine source, a range of synthetic methods were developed and the numerous new synthetic compounds were obtained [1,3,5,13,14,17,22–28].

## 2. Stereochemistry

Aplysinopsins with C-8-C-1′ double bonds can occur as two geometrical isomers. The ratio of *E/Z* isomers can be determined by NMR, based on a larger H-C(8),C(5′) ^1^H^13^C heteronuclear coupling constant in the *E* isomer, as compared to its *Z* counterpart [14]. These aspects of the aplysinopsins’ stereochemistry were comprehensively explored by Guella *et al.* [14,17]. All previous reports on aplysinopsins either did not consider geometric isomerism, or described stereoisomer *E* as privileged or exclusive.

Guella *et al.* [14] isolated 3′-deimino-3′-oxoaplysinopsin (**17**) from *Tubastrea* sp. scleractinian coral from the Philippines as a mixture of *E/Z* isomers (5:2). The synthesis of **17** via condensation of indole-3-carboxyaldehyde with 1,3-dimethylhydantoin resulted in a 95:5 mixture of *E:Z* isomers. Similar proportions were obtained for aplysinopsin (**1**) and 6-bromoaplysinopsin (**5**) after condensation of methylcreatinin and indole-3-carboxyaldehyde or 6-bromoindole-3-carboxyaldehyde respectively [14]. However, under the influence of UV irradiation, or even only under daylight, the mixture in the solution had undergone photoisomerization to become richer in the *Z* isomer [14].

From the scleractinian coral *Laptopsammia pruvoti,* 3′-deimino-2′,4′-bis(demethyl)-3′-oxo-aplysinopsin (**19**) and its brominated analogue (**20**) were isolated as a mixture *Z:E* isomers in the ratio 3:2 [14]. Interestingly, this time the synthesis of **19** gave predominantly the *Z* isomer. The authors concluded the lack of a methyl group at N2′ may be responsible for preferential formation of the *Z* isomer [14].

Crystal diffraction data for isomer *Z* of aplysinopsin (**1**) revealed that steric repulsions between H-C2 and Me-N2′ force the five-membered ring out of the plane of the indole nucleus, making N2′ tetrahedral [9]. Based on molecular mechanics calculations, an analogous conformation was proposed for the *Z* isomer of 3′-deimino-3′-oxoaplysinopsin (**17**) [14]. The authors concluded that owing to H-C2/Me-N2′ repulsions in the rate-limiting transition state of condensation/elimination leading to the *Z* isomer of that compound, the route to the *E* isomer, which avoids such repulsions, is followed. From the other side, the molecular mechanics calculation indicated the reverse situation for 3′-deimino-2′,4′-bis(demethyl)-3′-oxoaplysinopsin (**19**), in which N2′ bears an H-atom. The isomer *Z* was calculated to be less strained than isomer *E* owing to H-C2/C(5′)=O repulsions. This is reflected in the rate-limiting state of the condensation/elimination which leads preferably to the *Z* isomer of **19**, with gain of conjugation in the fully planar form [14]. An analogous explanation was offered for 6-bromo-3′-deimino-2′,4′-bis(demethyl)-3′-oxoaplysinopsin (**20**), isolated from *Laptopsammia pruvoti* as a 50:50 mixture of *E:Z* isomers. The synthesis produced almost exclusively the *Z* isomer (95%) that undergoes partial photoisomerization to isomer *E* [14].

In conclusion, aplysinopsin-type compounds without bulkier substituents at N2′ are formed predominantly in the *Z* configuration, whereas the opposite is true for the compounds bearing a methyl group at N2′.

The stereochemical outcome of the condensations leading to aplysinopsin synthesis is determined by the thermodynamic stability of the products. This was evaluated in studies on 6-bromo-4′-demethyl-3′-*N*-methylaplysinopsin (**9**) isolated from *Dendrophillia* sp. from the Philippines. This compound occurs naturally with isomers *Z:E* in a ratio 95:5 and easily undergoes photoisomerization to give a mixture enriched in the *E* isomer [17]. The latter reverted to the mixture of the original composition in a few days at room temperature in the dark, or after 2 h at 60°C. Therefore, the *Z* isomer of **9** was thermodynamically more stable, but photochemically more labile than its *E* one. This finding was confirmed by separation of isomers and evaluation of the ratio of their extinction coefficients at the irradiation wavelength of 350 nm. The ratio of ɛ*_Z_* to ɛ*_E_* was 2.5. The irradiation of pure *E* isomer for 2 h at room temperature led to a 1:3 *Z*/*E* mixture, whereas heating of neat *E* isomer at 60°C in the dark led to neat *Z* isomer [17]. For 3′-deimino-3′-oxoaplysinopsin (**17**) with a methyl group at N2′, the ratio of extinction coefficients ɛ*_Z_*/ɛ*_E_* equals 4. The irradiation of both pure isomers led to the mixture with composition *E*/*Z* = 3:1, as expected for a more extensive transformation of the isomer with stronger light absorption. When that mixture was heated under reflux in piperidine, the ratio changed until it reached 95:5 as observed for the synthetic compound [14].

In conclusion, it was shown that in the case of aplysinopsins with a methyl group at position 2′ (compounds **1**, **17**), isomer *E* is thermodynamically and photochemically more stable than isomer *Z*, while for the compounds with either an H-atom or a lone pair at N2′ (**3**, **4**, **9, 19**), isomer *Z* is thermodynamically more stable and photochemically more labile than isomer *E* [17].

The stereochemistry of aplysinopsins may have potential significance from a pharmacological point of view. For instance, the *E* isomer of methylaplysinopsin showed a stronger effect on neurotransmission than the *Z* isomer [9]. Therefore, the stereospecificity of the synthesis is of importance. On the other hand, thermostability and photostability could play a significant role in the ecology of the marine organisms containing aplysinopsin-type metabolites.

## 3. Biological Activity

Marine organisms have been confirmed to be a promising source of potentially valuable drugs. Aplysinopsins have aroused considerable interest as potentially useful medications. They show specific toxicity for cancer cells, as well as some antiplasmodial and antimicrobial activities. However, properties related to modulation of neurotransmission seem to be the most significant in the pharmacology of these compounds. Aplysinopsins have the potential to influence monoaminooxidase (MAO), and nitric oxide synthase (NOS) activities. They were also found to modulate serotonin receptors.

### 3.1. Neurotransmission

Methylaplysinopsin (**7**), isolated for the first time from the sponge *Aplysinopsin reticulate,* showed a potent antidepressant effect in mammals [9,29]. This activity was evaluated in tetrabenazine-induced ptosis in mice and rats. A pretreatment with methylaplysinopsin caused significant reduction in ptosis occurring in tetrabenazine treated rodents with ED_50_ 5 mg/kg [9,30]. Reversal of tetrabenazine ptosis has been also tested for other aplysinopsins [9]. Isoplysin (**2**) did not show activity, while aplysinopsin (**1**) and the analog **26** caused weak reduction of ptosis with ED_50_ values of 80 and 72 mg/kg, respectively [9].

An inhibition of monoamine oxidase (MAO), as well as an inhibition of the neuronal uptake of the neurotransmitters serotonin or noradrenalin, are common mechanisms accounting for the antidepressant effect [30]. In *in vitro* studies on mouse brain homogenate as a source of MAO, methylaplysinopsin exhibited enzyme inhibition over a wide concentration range (0.1–100 μM), especially with serotonin as a substrate. In addition, *ex-vivo* experiments with rats and mice, after oral administration of methylaplysinopsin, revealed MAO inhibition which is relatively short-term and reversible. The biological activity of that compound correlated also with its disappearance from the plasma. Pharmacokinetic studies on rats showed that after intravenous administration, methylaplysinopsin concentration in plasma declines rapidly over 4–8 h, reaching the level below 1 ng/mL 8 hours after dosing [30]. The relevant antidepressant activity achieved after intravenous and oral administration in mice suggested the presence of active metabolites of methylaplysinopsin. However, the comprehensive pharmacokinetics studies were limited because of sensitivity of HPLC detection of the parent compound [30].

The evaluation of the second possible mechanism responsible for antidepressant action showed that methylaplysinopsin enhanced serotonergic activity in the central nervous system. It inhibited the uptake of [^3^H]-serotonin into synapses prepared from the cerebral cortex. In addition, this compound amplified the release of [^3^H]-serotonin from prelabelled synaptosomes [30]. Kinetic studies on [^3^H]-serotonin - specific binding to a rat brain hippocampal membrane revealed that methylaplysinopsin was a relatively weak displacer of serotonin, with IC_50_ 160 μM (IC_50_ 16 nM was found for LSD). Methylaplysinopsin showed more potency in displacing [^3^H]-serotonin specifically bound to the crude homogenate of rat brain (IC_50_ value of 66 μM) [30]. The possible effects of methylaplysinopsin on uptake and release of noradrenalin were also evaluated and proved not significant [30]. In another study aplysinopsin (**1**) was tested in the Porsolt forced swim test and did not exhibit antidepressant activity [7].

In mammals there are six classes of G protein-coupled serotonin receptors [31]. They are localized in the cell membrane of nerve cells and other cell types and mediate the effect of serotonin and the endogenous ligand, and of a broad range of pharmaceutical drugs and hallucinogenic compounds. The aplysinopsins isolated from Jamaican sponge *Smenospongia aurea* were tested for affinity for human serotonin 5-HT_2_ receptor subtypes 5-HT_2A_ and 5-HT_2C_ expressed in a mammalian cell line [6]. Among others, the receptor 5-HT_2A_ participates in pathophysiology of depression [32], while 5-HT_2C_ regulates food intake in mammals [33]. Therefore, the modulation of these receptors could provide antidepressant and anti-obesity remedies, respectively.

The affinity constants to serotonin receptor subtypes 5-HT_2A_ and 5-HT_2C_ are presented in Table 2. Compounds **4**, **5** and **11** showed high affinity to the 5-HT_2C_ receptor. In addition, aplysinopsins **5** and **11** showed also affinity to the 5-HT_2A_ subtype. The highest affinity to 5-HT_2C_ was established for 6-bromoaplysinopsin (**5**), with a *K*i value similar to that of serotonin. Compounds **4** and **11** had only 4 to 20% of the activity of serotonin [6].

Two studied receptor subtypes 5-HT_2A_ and 5-HT_2C_ are genetically closely related, sharing highly conserved sequences (77%). In order to differentiate their functions, research is focused not only on affinity, but also on selectivity towards receptor subtypes. The compound **11** had no significant selectivity to studied receptors, and compound **5** had limited selectivity towards 5-HT_2C_ serotonin subtype (6-fold). Interestingly, 6-bromo-2′-de-*N*-methylaplysinopsin (**4**) showed significant selectivity to the 5-HT_2C_ serotonin subtype over the 5-HT_2A_ subtype [6].

The structure-activity relationship data revealed a significant role of X, R_1_, and R_2_ functional groups at positions 6, 2′ and 3′ in binding to human 5-HT_2_ receptors. The length of the alkyl chain at the R_2_, as well as bromination of position 6 of the indole ring increased the binding properties. In addition, the bromination also enhanced the selectivity to 5-HT_2C_ over 5-HT_2A_ subtype. Also, methylation at R_1_ position seems to facilitate binding to the 5-HT_2A_ receptor [6,34]. However, this analysis has to be yet verified, because it was performed on a limited number of analogs.

Another activity of aplysinopsin in neuromodulation is related to selective inhibition of nitric oxide synthase (NOS) [11]. Nitric oxide (NO) is an important secondary messenger with numerous functions such as regulation of blood pressure, inflammation, platelet adhesion, neurotransmission and defense mechanisms. NO is a reactive molecule with one unoccupied electron, therefore its excessive production causes many diseases, e.g. postischemic stroke damage, schizophrenia, colitis, tissue damage and pathological inflammation. The biosynthesis of NO is catalyzed by nitric oxide synthase (NOS), which occurs in three isoforms: inducible NOS (iNOS), endothelial NOS (eNOS), and neuronal NOS (nNOS). nNOS and eNOS are constitutive Ca^2+^/calmodulin-regulated enzymes, whereas iNOS is a Ca^2+^/calmodulin-independent enzyme induced in macrophage. Selective inhibitors of each NOS isoenzyme have potential as therapeutic agents. A bioassay guided fractionation of extract isolated from sponge *Hyrtios erecta* led to isolation of aplysinopsin-type compounds with selective inhibitory activity towards nNOS [11]. The screening was performed on iNOS prepared from mouse macrophage and nNOS obtained from rat cerebrum. AcOEt extract showed 94% inhibition against nNOS and only 21% inhibition against iNOS at 125 μg/mL. The active compounds were identified as 5,6-dibromo-2′-demethylaplysinopsin *Z*-**10** (100% inhibition of nNOS at 125 μg/mL and 32% inhibition at 25 μg/mL), isomer *E*-**10** (100% inhibition of nNOS at 125 μg/mL and 13% inhibition at 25 μg/mL) and isomer *Z*-**4** with 100% inhibition of nNOS at 125 μg/mL and 22% inhibition at 25 μg/mL. *N*-monomethyl-L-arginine (L-NMMA), a known NOS inhibitor, showed 60% inhibition of nNOS at 25 μg/mL. While L-NMMA inhibited iNOS with the same potency, aplysinopsins showed high selectivity towards nNOS. 5,6-Dibromo-2′-demethylaplysinopsin (**10**) isomers did not affect iNOS at all, and 6-bromo-2′-demethylaplysinopsin (**4**) inhibited only 7.5% of iNOS activity at a concentration of 125 μg/mL [11].

### 3.2. Antineoplastic Activity

An antitumor action was the first bioactivity described for aplysinopsins. As a matter of fact, bioassay guided fractionation of the sponge *Verongia spengelli* resulted in isolation of aplysinopsin (**1**) as the factor that showed inhibitory activity *in vivo* against P388 lymphocytic leukemia in mice (T/C, tumor volume treated group/tumor volume control group × 100% equal 135 at 200 mg/kg) [2]. Aplysinopsin also appeared to be cytotoxic against P338, human epidermoid carcinoma KB and murine lymphoma LH-1220 cancer cell lines [2]. In another study, isoplysin (**2**) was found to inhibit LH-1220 (IC_50_ 11.5 μg/mL) and KB (31% inhibition at 20 μg/mL) cell growth. In addition, aplysinopsin (**1**) and methylaplysinopsin (**7**) showed cytotoxicity against LH-1220 (IC_50_ 2.3 and 3.5 μg/mL respectively), and against KB (IC_50_ 3.5 and 6.7 μg/mL respectively) [10].

In general, because of its relatively low potency in comparison with other anticancer agents, this activity of aplysinopsins has not been of major interest. Conversely, only a few basic compounds from a variety of aplysinopsins isolated from Nature have been screened.

### 3.3. Antiplasmodial Activity

A group of aplysinopsins isolated from the sponge *Smenospongia aurea* (**2–5**,**7**, and **11**) were tested against a D6 clone of *Plasmodium falciparum* for their *in vitro* antimalarial activity [6]. The highest activity was found for 6-bromoaplysinopsin (**5**) at 0.34 μg/mL with selectivity index 14 (S.I. = IC_50_ [Vero cells)/IC_50_ (*P. falciparum*)]. However, it proved inactive in *in vivo* studies. Isoplysin (**2**) and 6-bromo-2′-de-*N*-methylaplysinopsin (**4**) showed moderate activity at 0.97 and 1.1 μg/mL with selectivity index (S.I) > 4.9 and > 4.3 respectively. In addition, 6-bromo-2′-de-*N*-methylaplysinopsin (**4**) inhibited the antimalarial target plasmepsin II enzyme with IC_50_ 53 μM (FRET) and 66 μM (FP) [6].

### 3.4. Antimicrobial Activity

The presence of antimicrobial substances in marine organisms has been described as a common phenomenon. Several researchers have also evaluated antimicrobial properties of aplysinopsins. In the first report, a mixture of two compounds, 6-bromoaplysinopsin (**5**) and 6-bromo-4′-de-*N*-methyl-aplysinopsin (**6**) showed a weak inhibition zone for *Bacillus subtillis* and no activity against *E. coli, S. cerevisiae,* and *Penicillium atrovenetum* [5]. In another study, aplysinopsin (**1**) and 6-bromo-aplysinopsin (**5**) were screened for a range of antibacterial, antifungal and antiviral activities. The only activity detected was growth inhibition of the fungus *Trichophyton mentagrophytes* by aplysinopsin (**1**) [35].

Another investigation involved screening of an extract from the Australian scleractinian coral *Tubastreae faulkneri* for activity against microorganisms isolated from local waters: *Vibrio alginolyticus, V. harveyi, V. parahaemolyticus, Photobacterium damsela, Alteromonas rubra, Synechococcus* sp. and *Staphylococcus aureus.* These microbes were possibly encountered by *Tubastreae faulkneri* and the evolution of defensive chemical mechanisms could be expected. The methanol extract of the coral showed an inhibitory activity towards all microbes, besides *V. parahaemolyticus* [18]. The authors found that the methanolic fraction of the coral consisted of 72% aplysinopsin-like compounds (**5**, **4** and dimer **27**). Antimicrobial assays revealed that all of these compounds were active against *Synechococcus* sp. In addition, **1** and **5** slightly inhibited the growth of *Staphylococcus aureus* [18]. Finally, aplysinopsins isolated from Indonesian reef sponges *Thorectandra sp* and *Smenospongia* sp. (**12**, **13**, **14**, **15**, and **16**) were tested against *Staphylococcus epidermis* [12]. They showed weak to moderate toxicity with minimum inhibitory concentration (MIC) ranging from 6.25–100 μg/mL as compared to standard vancomycin (0.625 μg/mL) [12].

## 4. Ecological Significance

Aplysinopsins belong to the group of secondary metabolites that do not participate in primary functions of the organisms and are therefore considered not necessary for their survival. Therefore, the investment of resources in the synthesis of these energy-costly compounds implies their importance in interactions of marine organisms with the biotic and/or abiotic environment [36]. Several possible functions of aplysinopsins could be predicted from a few ecological studies. In addition, bioactivity of these compounds described in the previous chapter could reflect their role for marine organisms.

The biogenetic origin of aplysinopsins should be the first concern in consideration of their ecological significance. Initially, aplysinopsins were believed to be exclusive to marine sponges. They were proposed as the chemotaxonomic marker for a separate genus *Aplysinopsis*. Nevertheless, chemotaxonomic data were sometimes inconsistent, and sometimes distinct species contained the same group of compounds [4,7,15]. In addition, aplysinopsins were isolated from many species of coral (Table 1), as well as from sea anemone [20] and nudibranch *Phyllida melanobrachia* [15]. The multiple sources of aplysinopsins may suggest their common microbial origin. However, no metabolic pathway of aplysinopsins has been established so far.

One possible function of aplysinopsins, resulting from their photoisomerization, could be protection against excessive UV radiation. It is known that coral reef fauna of the shallow-water of the tropics can be damaged by direct sun radiation, and only species protected by pigments can survive in such areas. Photoisomerization could be a non-destructive process for entrapping radiant energy that protects invertebrates from sun burning. Unfortunately, aplysinopsin-type compounds easily undergo photoisomerization even under the light condition of the laboratory; therefore neither the (*E*)/(*Z*) stereoisomeric ratio for the aplysinopsins in the nature, nor its possible dependence on the wavelength of solar radiation filtered by the sea, would be easy to assess [14].

Like many other compounds isolated from marine organisms, aplysinopsins could also play a role in an antimicrobial defense. However, screening for growth inhibition of various microorganisms did not show a potent activity (see description in Biological Activity section). Only one study confirmed their possible action in protection against microbial infections [18]. In that study, aplysinopsins extracted from coral *Tubastreae faulkneri* were evaluated against microorganisms isolated from its natural environment. Therefore, in response of previous exposure to these organisms, the coral could evolve defensive mechanisms.

An interesting ecological case has been described for a nudibranch *Phyllida melanobrachia* which feeds exclusively on the coral *Tubastrea coccinea* [15]. Both of these organisms show cryptically colored orange pigmentation, with aplysinopsins as major constituents. Interestingly, the mollusk seemed to be selective in retention of certain metabolites from the coral. Two aplysinopsin derivatives **3** and **4,** were found in extracts from both *P. melanobrachia* and *T. coccinea.* In turn, compounds **5**, **12** and **13** were isolated exclusively from the coral [15]. The authors proposed the hypothesis that the mollusk accumulates aplysinopsins from the coral that it feeds on as a defensive strategy against its own predators. A similar adaptation mechanism has been proved earlier for other invertebrates that lack physical protection. However, the role of aplysinopsins for *Phyllida melanobrachia* has not been further investigated.

Another attempt to establish an ecological role for aplysinopsins was made by Fusetani *et al.* [16]. The object of their study was a scleractinian coral *Tubastrea aurea*, a common species in temperate and tropical waters. It possesses well-developed polyps with colors ranging from yellow to reddish-orange. The ethanol extract of *T. aurea* inhibited the cell division of fertilized sea urchin. Aplysinopsin (**1**), which appeared to be responsible for the activity, reached 1.2 mg/g of the polyp’s wet weight, and inhibited the first cleavage of fertilized sea urchin eggs at 2.5 μg/mL. Since the color of the polyps is most likely attributable to aplysinopsins, it is possible that they are also responsible for a defense from predators. However, no ichthyotoxic activity towards *Oryzias latipes* was found [16].

A comprehensive study on an ecological function of aplysinopsins was provided by Koh and Sweatman [18]. They proved that aplysinopsins extracted from the coral *Tubastreae faulkneri* inhibited larval growth of competitive coral species. The intense competition for space among sessile organisms is often manifested as overgrowth of competitive individuals and epibiosis. These species need specific conditions for their habitat. They require the access to an unobstructed water flow to obtain food, as well as access to sunlight for their symbiotic algae. One survival strategy is resource allocation during a rapid growth; the other is an evolution of defensive mechanisms that would compensate for slow growth and provide resistance against invasion of the faster growing organisms. Such mechanisms involve the use of protective organs, e.g. spins, sweeper tentacles, mesenterial filaments, and/or synthesis of defensive compounds, which could act as either repellents or toxins [18].

*Tubastreae faulkneri* is an Australian scleractinian coral of the family Dendrophylliidae. It was found to inhabit overhangs and vertical surfaces in the depth range 3–7 m. This coral, which is a slow growing species, shares the shallower parts of its range with fast-growing species. Koh and Sweatman [18] established that the ethanol extract of *Tubastreae faulkneri* consisted of aplysinopsins (**1**, **5**, **4** and dimer **27**). For the study on the chemical defense involved in persistence of *Tubastreae faulkneri*, eleven species of scleractinian corals were selected regarding their competitive abilities and a range of habitat. These included members of four families: Acroporidae: *Montipora digitata*, *Acropora formosa*, *A. millepora*, *A. pulchra*, *A. tenuis*, Faviidae: *Favia pallida*, *Gonisterea aspera*, *Platygyra daedalea*, *P. sinensis*, Fungiidae: *Fungia fungites*, and Pectiniidae: *Oxypora lactera*. The test was also performed on the self larvae of *Tubastreae faulkneri.* The toxicity of *Tubastreae faulkneri* extracts to scleractinian planulae was determined for a range of concentrations that were 100 or more below the concentration of *Tubastreae faulkneri* tissues (50, 100, and 200 μg/mL of sea water). The proportion of dead planula was taken as an indicator of toxicity.

Among all larvae of 12 coral species tested, the self larva of *Tubastreae faulkneri* was the only one that did not suffer toxic effects after exposure to extracts from adult *Tubastreae faulkneri.* Larvae of the rest of the species suffered high mortality at all tested concentrations of *Tubastreae faulkneri* extract. The most sensitive were *Oxypora lactera* and *Platygyra daedalea,* with toxic effects effective at only 10 μg/mL. The authors concluded that such a broad spectrum of activity affecting 11 species from 4 genera of coral may indicate that the extract of *Tubastreae faulkneri* consists predominantly of aplysinopsins, and plays significant ecological roles in eliminating potential competitors. This seemed to be reflected in Nature, because two most sensitive species have been never observed in the vicinity of *Tubastreae faulkneri,* despite sharing the same habitat preferences [18].

This interesting phenomenon, that conspecific larvae were not susceptible even to the highest concentrations of *Tubastreae faulkneri*, could indicate the specific adaptation to high doses of adult metabolites, since the larvae are broaded within the adult during embryogenesis. Therefore, these larvae should be able to settle near adults without harm and at the same time not face competition from other coral recruits. In fact, it has been observed that larvae of *Tubastreae faulkneri* settle preferentially in the presence of adult colonies [18].

Insolubility in water may increase efficiency of aplysinopsins in preventing neighboring competitors from occupying the space. Once secreted by the coral, aplysinopsins could be bound selectively on organic films and surfaces in the vicinity of the coral and not to be washed away. Such strategy decreases the costs of maintaining the same level of chemicals over time, in contrast to secretion of water soluble compounds, which diffuse easily from the source [18].

Probably the most fascinating example of aplysinopsins’ function is their involvement in the induction of symbiosis between anemonefish and the tropical sea anemone that host them [20]. This extreme specialization involves 28 species of coral reef fish belonging to the family Pomacentridae, all characterized by living in symbioses with sea anemones of several genera. Some anemonefish cooperate only with a single or a few species of sea anemone, being immune to their poisonous tentacles, but sensitive to those of other species of sea anemone. Anemonefish have a limited capability of surviving predation when away from their sea anemone host [37]. After the planktonic period, juvenile fish enter the benthic life searching for their partner anemone to begin the symbiosis. Naive juveniles of anemonefish are innately protected from the fatal sting of their sea anemone host. However, the mechanisms of specific partner recognition leading to species-specific symbiosis still remain unclear. Visual cues did not play an important role in that process, but the fish rather recognize their partners from chemicals secreted by the anemone. An experiment on *Radianthus kuekenthali* sea anemone and *Amphiprion perider* anemonefish proved for the first time that aplysinopsins can be listed among chemicals responsible for symbiosis establishment. The juvenile fish were kept in acrylic vessels supplied with seawater from one end. Individual samples of chemicals isolated from anemone *Amphiprion perider* were dissolved in seawater and added dropwise from the upstream end. Active upstream and downstream movements of fish, so called ‘attractive swimming’ towards the sample indicated sensitivity to chemical stimulus. While control seawater or an inactive fraction was administrated, the fish stayed at the starting point. Bioactivity guided fractionation of anemone extract active in the active swimming test led to isolation of aplysinopsin-type compounds. The dihydroaplysinopsins elicited active swimming at a concentration of 10^−6^M, whereas at the same concentration aplysinopsin derivatives with a C-8 – C-1′ double bond, induced the fish to perform ‘seesawing’, a head up and down movement behavior characteristic in nature [20]. On the other hand, aplysinopsins did not exhibit synomonal activity in other species of fish sharing the same host. That could indicate that two different species of symbiotic anemonefish recognize their common host through different chemicals [20].

Another possible function of aplysinopsins could be extrapolated from the studies performed by Lindel *et al.* [28] on inhibition of fish feeding by compounds derived from sponge and its synthetic analogues. Sponges can be a rich protein diet source and their soft body lacking physical protection is easily vulnerable to intense grazing by fishes. The test on anti-feeding activity of compounds structurally similar to aplysinopsins showed that pyrrole-imidazole alkaloids significantly inhibited consumption of a studied fish *Thalassoma bifasciatum* [36].

## 5. Summary

Despite many diverse reports on biological activity of aplysinopsin-type compounds, their potential has never been exploited comprehensively. Most of the single studies evaluated a limited number of compounds isolated from natural sources and activities were not verified for a sufficient number of other analogues to allow for valuable conclusions or for analysis of structure-activity relationships. One explanation for this is limited efficiency of isolation from natural sources. In addition, limited solubility in most organic solvents and insolubility in water, together with relatively high polarity, do not encourage studies on these molecules.

Our review summarizes all information on bioactivity of aplysinopsins and hopefully supports the need for further research on these interesting molecules. Some valuable conclusions can be acquired both from pharmacological and ecological studies performed in the past. First of all, aplysinopsins are able to inhibit cell division, which is reflected in their anticancer actions. However, for marine organisms themselves these properties help to inhibit growth of competitors, as showed in case of coral *Tubastreae faulkneri* and larvae of neighboring corals.

The most potent pharmacological activity of aplysinopsins is related to modulation of the central nervous system. This can be explained by possible roles they play for marine organisms in inhibition of predators’ feeding activity (affinity to the 5-HT_2C_ receptor that regulate food intake, inhibition of feeding of fish); sedation of predators (affinity to the 5-HT_2A_ receptor); as well as regulation of symbiosis establishment as in the case of a sea anemone (stimulation of anemone fish behavior). All the above benefits offer an evolutionary explanation for the metabolic costs involved in synthesis of aplysinopsins, if these compounds are synthesized by organisms that accumulate them in tissues (sponges, corals, sea anemone). The other option is that aplysinopsins are synthesized by associated microbes. Then, the advantageous actions of these compounds for sea invertebrates are included in the costs of maintenance of symbiotic microorganisms.

## Figures and Tables

	Compound	X	Y	R_1_	R_2_	R_3_

**1**	aplysinopsin	H	H	CH_3_	H	CH_3_
**2**	isoplysin A	H	H	CH_3_	CH_3_	H
**3**	2′-de-*N*-methyl-aplysinopsin	H	H	H	H	CH_3_
**4**	6-bromo-2′-de-*N*-methylaplysinopsin	Br	H	H	H	CH_3_
**5**	6-bromoaplysinopsin	Br	H	CH_3_	H	CH_3_
**6**	6-bromo-4′-de-*N*-methylaplysinopsin	Br	H	CH_3_	H	H
**7**	methylaplysinopsin	H	H	CH_3_	CH_3_	CH_3_
**8**	4′-demethyl-3′-*N*-methylaplysinopsin	H	H	H	CH_3_	CH_3_
**9**	6-bromo-4′-demethyl-3′-*N*-methyl-aplysinopsin	Br	H	H	CH_3_	CH_3_
**10**	5,6-dibromo-2′-demethylaplysinopsin	Br	Br	H	H	CH_3_
**11**	*N*-3′-ethylaplysinopsin	H	H	CH_3_	CH_2_CH_3_	CH_3_

	Compound	X	R

**12**	1′,8-dihydroaplysinopsin	H	H
**13**	6-bromo-1′,8-dihydro-aplysinopsin	Br	H
**14**	6-bromo-1′-hydroxy-1′,8-dihydroaplysinopsin	Br	OH
**15**	6-bromo-1′-methoxy-1′,8-dihydroxyaplysinopsin	Br	OCH_3_
**16**	6-bromo-1′-ethoxy-1′,8-dihydroxyaplysinopsin	Br	OCH_2_CH_3_

	Compound	X	R_1_	R_3_

**17**	3′-deimino-3′-oxo-aplysinopsin	H	CH_3_	CH_3_
**18**	6-bromo-3′-deimino-3′-oxoaplysinopsin	Br	CH_3_	CH_3_
**19**	3′-deimino-2′,4′-bis(demethyl)-3′-oxo-aplysinopsin	H	H	H
**20**	6-bromo-3′-deimino-2′,4′-bis(demethyl)-3′-oxoaplysinopsin	Br	H	H

	Compound	X	Z	R_2_
**21**	N-propionylaplysinopsin	H	OCCH_2_CH_3_	H
**22**	6-bromo-N-propionylaplysinopsin	Br	OCCH_2_CH_3_	H
**23**	N-methylaplysinopsin	H	CH_3_	H

**Table 1:** The source and activity of aplysinopsins isolated from Nature.

	Source	Activity
**1**	*Thorecta sp.* sponge Great Barrier Reef Australia [1] *Verongia spengelli* sponge Florida Keys [2] *Dercitus* sp. sponge Caribbean [3] *Smenospongia aurea* sponge Caribbean [5] *Dictyoceratida* sponges [8] *Astroides calycularis* anthozoan Mediterranean [13] *Tubastraea aurea* Japan scleractinian coral [16] *Tubastraea* sp. scleractinian coral Philippines [14] *Radianthus kuekenthali* sea anemone Japan [20] *Aplysina* sp. sponge Japan [10] *Tubastraea faulkneri* scleractinian coral Australia [18] *Tubastraea* sp. scleractinian Japane [19] *Smenospongia* sp. sponge Indo-Pacific reefs [12] *Verongula rigida* sponge Florida Keys [7]	Anticancer [2,10] Antimicrobial [12] Inhibitor of development of fertilized sea urchin eggs [16] Induces symbiosis between sea anemone and anemone fish [20]
**2**	*Aplysina* sp. sponge Japan [10] *Smenospongia aurea* sponge Jamaica [6]	Anticancer [10] Antiplasmodial [6]
**3**	*Dercitus* sp. sponge Caribbean [3] *Tubastrea coccinea* coral Hawaii [15] *Phestilla melanobrachia* mollusc [15] *Dendrophyllia* sp. scleractinian coral Philippines [17] *Smenospongia aurea* sponge Jamaica [6] *Verongula rigida* sponge Florida Keys [7]	
**4**	*Dercitus* sp. sponge Caribbean [3] *Tubastrea coccinea* coral Hawaii [15] *Phestilla melanobrachia* mollusc [15] *Dendrophyllia* sp. scleractinian coral Philippines [17] *Tubastraea faulkneri* scleractinian coral Australia [18] *Smenospongia aurea* sponge Jamaica [6] *Hyrtios erecta* sponge Japan [11]	Serotonin receptors modulator [6] Antiplasmodial [6] Inhibitor of nitric oxide synthase (nNOS) [11]
**5**	*Tubastrea coccinea* coral Hawaii [15] *Smenospongia aurea* sponge Caribbean [5] *Astroides calycularis* anthozoan Mediterranean [13] *Radianthus kuekenthali* sea anemone Japan [20] *Tubastraea faulkneri* scleractinian coral Australia [18] *Smenospongia aurea* sponge Jamaica [6] *Smenospongia aurea* sponge Florida Keys [7]	Serotonin receptors modulator [6] Antiplasmodial [6] Induces symbiosis between sea anemone and anemone fish [20]
**6**	*Smenospongia aurea* sponge Caribbean [5]	
**7**	*Aplysinopsis reticulata* sponge Australia [9] *Smenospongia aurea* sponge Jamaica [6]	Antidepressant – inhibitor of monoamine oxidase [9,29,30]
**8**	*Dendrophyllia* sp. scleractinian coral Philippines [17] *Smenospongia aurea* sponge Jamaica [6]	
**9**	*Dendrophyllia* sp. scleractinian coral Philippines^17^	
**10**	*Hyrtios erecta* sponge Japan [11]	Inhibitor of nitric oxide synthase (nNOS) [11]
**11**	*Smenospongia aurea* sponge Jamaica [6]	Serotonin receptors modulator [6]
**12**	*Tubastrea coccinea* coral Hawaii [15] *Radianthus kuekenthali* sea anemone Japan [20] *Thorectandra* sp. sponge Indo-Pacific reefs [12]	Induces symbiosis between sea anemone and anemone fish [20]
**13**	*Tubastrea coccinea* coral Hawaii [15] *Radianthus kuekenthali* sea anemone Japan [20] *Thorectandra* sp. sponge Indo-Pacific reefs [12]	Antimicrobial [12]
**14**	*Thorectandra* sp. sponge Indo-Pacific reefs [12]	Antimicrobial [12]
**15**	*Thorectandra* sp. sponge Indo-Pacific reefs [12]	Antimicrobial [12]
**16**	*Smenospongia sp.* sponge Indo-Pacific reefs [12]	Antimicrobial [12]
**17**	*Thorecta sp.* sponge Great Barrier Reef Australia [1] *Tubastraea* sp. scleractinian coral Philippines [14]	
**18**	*Astroides calycularis* anthozoan Mediterranean [13] *Tubastraea* sp. scleractinian coral Philippines [14]	
**19**	*Leptopsammia pruvoti* scleractinian coral France [14]	
**20**	*Smenospongia aurea* sponge Caribbean [4] *Leptopsammia pruvoti* scleractinian coral France [14]	
**21**	*Astroides calycularis* anthozoan Mediterranean [13]	
**22**	*Astroides calycularis* anthozoan Mediterranean [13]	
**23**	*Aplysina* sp. sponge Japan [10]	
**24**	*Tubastraea faulkneri* scleractinian coral Australia [18]	Antimicrobial [18]
**25**	*Tubastraea* sp. stony coral Japan [19]	
**26**	*Tubastraea* sp. stony coral Japan [19]	
**27**	*Tubastraea* sp. stony coral Japan [19]	

**Table 2:** Affinity of aplysinopsins for human serotonin 5-HT_2_ receptors (equilibrium affinity constant) (after [6], modified).

aplysinopsin	5-HT_2A_ (*K*i, μM)	5-HT_2C_ (*K*i, μM)	Selectivity towards 5-HT_2C_
isoplysin A (2)	NA	NA	--
2′-de-*N*-methylaplysinopsin (2)	NA	NA	--
6-bromo-2′-de-*N*-methyl-aplysinopsin (6)	>100	2.3	>43
6-bromoaplysinopsin (5)	2.0	0.33	6
methylaplysinopsin (7)	NA	NA	--
*N*-3′-ethylaplysinopsin (11)	1.7	3.5	0.5
*serotonin*	*0.32*	*0.13*	
